# Transcriptome analysis of genes involved in starch biosynthesis in developing Chinese chestnut (*Castanea mollissima* Blume) seed kernels

**DOI:** 10.1038/s41598-021-82130-6

**Published:** 2021-02-11

**Authors:** Lingling Shi, Jia Wang, Yujun Liu, Chao Ma, Sujuan Guo, Shanzhi Lin, Jianzhong Wang

**Affiliations:** Beijing Advanced Innovation Center for Tree Breeding By Molecular Design, College of Biological Sciences and Biotechnology, National Engineering Laboratory for Tree Breeding, Beijing Forestry University, Beijing, 100083 China

**Keywords:** Molecular biology, Plant sciences

## Abstract

Chinese chestnut (*Castanea mollissima* Blume) seed kernels (CCSK) with high quality and quantity of starch has emerged as a potential raw material for food industry, but the molecular regulatory mechanism of starch accumulation in developing CCSK is still unclear. In this study, we firstly analyzed the fruit development, starch accumulation, and microscopic observation of dynamic accumulation of starch granules of developing CCSK from 10 days after flowering (DAF) to 100 DAF, of which six representative CCSK samples (50–100 DAF) were selected for transcriptome sequencing analysis. Approximately 40 million valid reads were obtained, with an average length of 124.95 bp, which were searched against a reference genome, returning 38,146 unigenes (mean size = 1164.19 bp). Using the DESeq method, 1968, 1573, 1187, 1274, and 1494 differentially expressed unigenes were identified at 60:50, 70:60, 80:70, 90:80 and 100:90 DAF, respectively. The relationship between the unigene transcriptional profiles and starch dynamic patterns in developing CCSK was comparatively analyzed, and the specific unigenes encoding for metabolic enzymes (SUSY2, PGM, PGI, GPT, NTT, AGP3, AGP2, GBSS1, SS1, SBE1, SBE2.1, SBE2.2, ISA1, ISA2, ISA3, and PHO) were characterized to be involved potentially in the biosynthesis of G-1-P, ADPG, and starch. Finally, the temporal transcript profiles of genes encoding key enzymes (*susy2*, *pgi2*, *gpt1*, *agp2*, *agp3*, *gbss1*, *ss1, sbe1, sbe2.1, sbe2.2, isa1, isa2, isa3*, *and pho*) were validated by quantitative real-time PCR (qRT-PCR). Our findings could help to reveal the molecular regulatory mechanism of starch accumulation in developing CCSK and may also provide potential candidate genes for increasing starch content in Chinese chestnut or other starchy crops.

## Introduction

Chinese chestnut (*Castanea mollissima* Blume), a deciduous tree in the family *Fagaceae* and genus *Castanea*, is a chestnut variety of considerable economic importance that is widely distributed in temperate and subtropical regions of the Northern Hemisphere^1–3^. In China, the chestnut has been cultivated for more than 3000 years, currently covering a total area of about 335,904 ha, and Chinese annual production (1.94 million tons) accounts for about 83.34% of global chestnut production (2.33 million tons)^4^. Most importantly, chestnut has high nutritional value, containing essential fatty acids, minerals (K, P, and Mg), vitamins, dietary fiber, and amino acids^5^, and is thus considered the king of dried fruits. In addition to direct consumption, chestnut is also used in the food processing industry, such as in *marron glace* production, chestnut flour for bread-making, and confectionery paste for desserts and jams^6–8^. Chestnut is a major arboreal nut crop and its chemical composition reveals that starch is the most abundant component, comprising approximately 47–80% of the dry kernel weight^9,10^, differing significantly from other temperate seeds, which are oily^11^. This starchy property also makes Chinese chestnut a potential substrate for fermentation, which could be developed for biological production in addition to utilization in the food industry.

Starch, as the main storage carbohydrate in vascular plants, is the most important dietary source of energy for humans, accounting for 80% of daily caloric intake, and thus plays an essential role in the food industry^12–14^, while also acting as a sustainable feedstock for numerous industrial applications^15^. There is an increasing industrial demand for starches with a global demand of 180 million tons per year^16^, hence different alternative sources of starches with high quality and quantity should be explored. In recent years, forestry biomass energy has gained attention from all sectors of society due to its remarkable characteristics of being “green, low-carbon and sustainable”^17–19^. Starch energy is a major resource for forestry biomass energy, which has been studied mainly in *Quercus*, a genus of the *Fagaceae* family, for sustainable forest biomass production^20^. Chinese chestnut, which belongs to the same family as *Quercus*, is well known as a food product and also a potential bioenergy resource or alternative raw materials for industry due to its starchiness. Recent researches reported that isolated starches from chestnut fruits can be an attractive source to formulate hydrogels with different mechanical and suitable physicochemical properties, which would be a valorization of industrial chestnut by-products^21,22^. The modified starch, carboxymethyl chestnut starch (CMCS) can be applied in the industrial processing of paste foods and frozen food as a thickening agent^23^. In addition, recent researches on chestnut starch have investigated its characteristics for industrial utilization, chemical composition, isolation methods, functional properties, physicochemical characteristics, modification of its structure and digestibility^7,9,10,24,25^, as well as its morphology and starch characteristics with different cooking styles^26^, but little is known about the synthesis of chestnut starch. Thus, it is essential to investigate the regulatory mechanisms of starch biosynthesis in chestnut to improve its starch content and extend its application in food industry.

Recently, Illumina RNA sequencing (RNA-Seq) technology has been used to investigate gene expression in various plant tissues at different developmental stages^27–30^. Although a Chinese chestnut reference genome has been published, its annotation remains incomplete. RNA-Seq analysis of developing CCSK could support a more comprehensive and reliable genetic information database, which could be used for research into chestnut molecular breeding to promote the development of chestnut resources for food, forest biomass energy or industrial material.

Herein, fruit development was surveyed and kinetic changes of starch granules were observed using a microscope during growth period (10, 30, 40, 50, 60, 70, 80, 90, and 100 days after flowering, DAF). In addition, dynamic patterns of sucrose, starch and its components (amylopectin and amylose) were measured at different developmental stages (50, 60, 70, 80, 90, and 100 DAF). Thus, the major periods of starch biosynthesis, which were optimal for transcriptomic analysis, were determined in CCSK. Transcriptome sequencing was carried out using Illumina-Solexa technology, and the obtained unigenes were functionally annotated. Furthermore, differentially expressed genes for key enzymes involved in starch synthesis in CCSK were screened using the DESeq method, and the results were verified via qRT-PCR. Overall, this study presents a systematic analysis for dynamic pattern of starch accumulation and transcript profiles of metabolic enzymes involved in starch biosynthesis in developing CCSK, which will contribute to clarifying the mechanisms of starch accumulation, thus supporting future work to increase starch biosynthesis and accumulation in CCSK and to extend the application of chestnut starch in industry.

## Results and discussion

### Characteristics of fruit growth and starch granule accumulation in developing Chinese chestnut fruits

**Figure 1:**
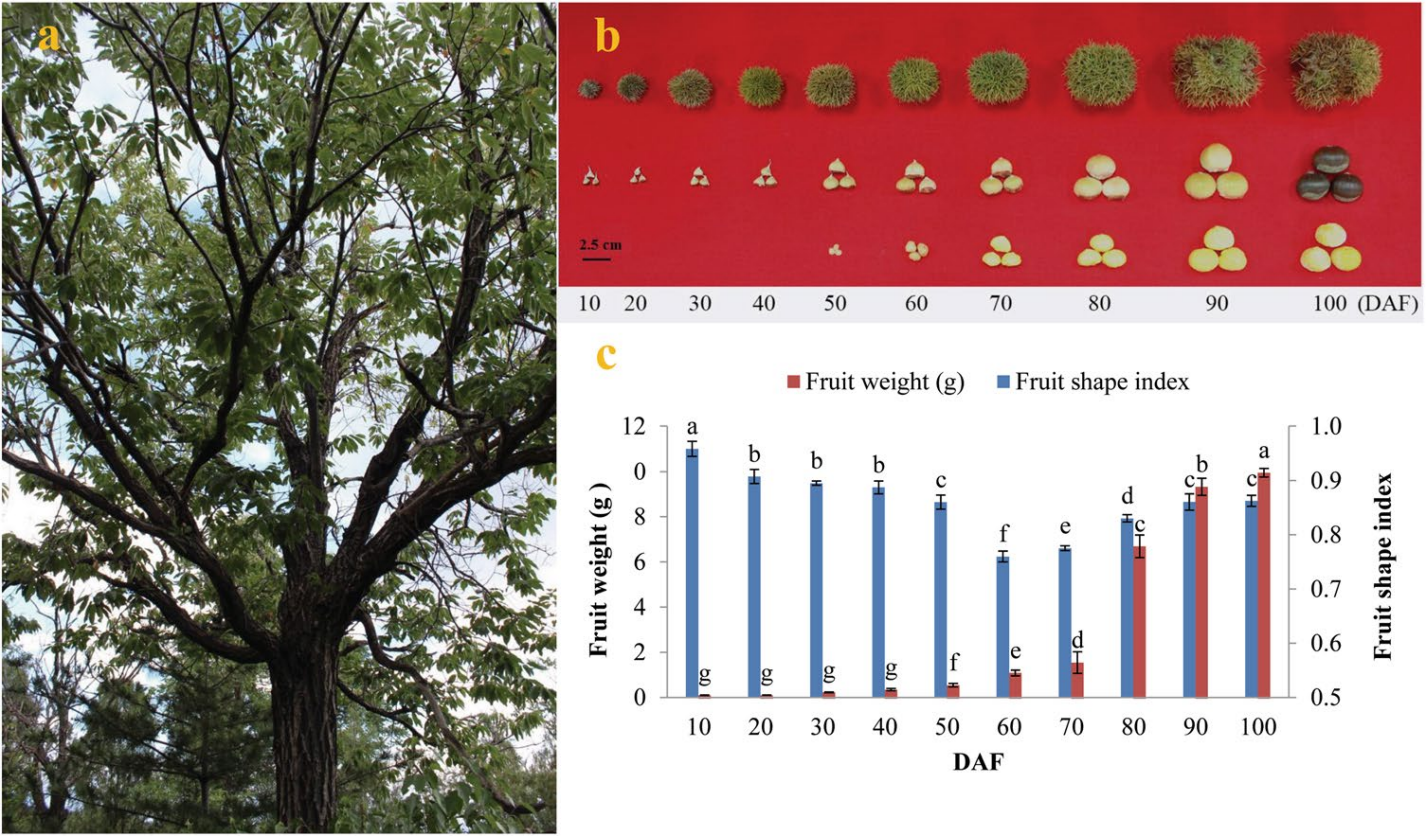
Features of *Castanea mollissima* fruits. (**a**) *Castanea mollissima* during fruiting. (**b**) Features of fruits at different developmental stages. (**c**) Fresh weight and fruit shape index (ratio of longitudinal and transverse diameters) of developing fruits. Error bars display SD, n = 3.

Application of chestnut starch in product development and industrial processing is guided by its end use properties such as composition, physicochemical and functional properties. Thus, to obtain high quality and high quantity chestnut starch and widen its application in food industry, the accumulation characteristics of starch should be surveyed in developing CCSK. Since starch is the main component of fruit, the growth and development of chestnut fruit was elaborated firstly. Chestnuts were observed throughout the entire growth period (during 10–100 DAF). We found that 2–4 nuts were contained within the spherical spiked involucre, which was up to 11 cm in diameter at maturity. The nuts were pale yellow during the early growth period and gradually darkened to dark brown. After removal of the chestnut shell, no seed kernels were observed during development until 50 DAF, and the kernel color gradually changed from the initial white to yellow during fruit ripening (Fig. 1a, b). To explore the dynamic growth patterns of fruits during development, we analyzed the growth tendency (fruit shape index and fresh weight) throughout the developing period of 10–100 DAF for the first time in Chinese chestnut. Here, the fruit shape index showed a significant decrease from 10 DAF (0.96 ± 0.01) to 60 DAF (0.76 ± 0.01), and then increased to 0.86 ± 0.01 at 100 DAF, revealing that fruit shape changed from the initial oval to hemispheric during development (Fig. 1b, c), which corresponded to the previous study on *C. sativa*^9^. We also noticed that the fresh weights of developing fruits gradually increased with a significant increase (6.1-fold) from 70 DAF (1.54 ± 0.07 g) to 90 DAF (9.33 ± 0.03 g), followed by a slight increase (10.9%) at 100 DAF (9.95 ± 0.03 g) (Fig. 1c), indicating that the most rapid growth of fruits occurs during 70–90 DAF.

**Figure 2:**
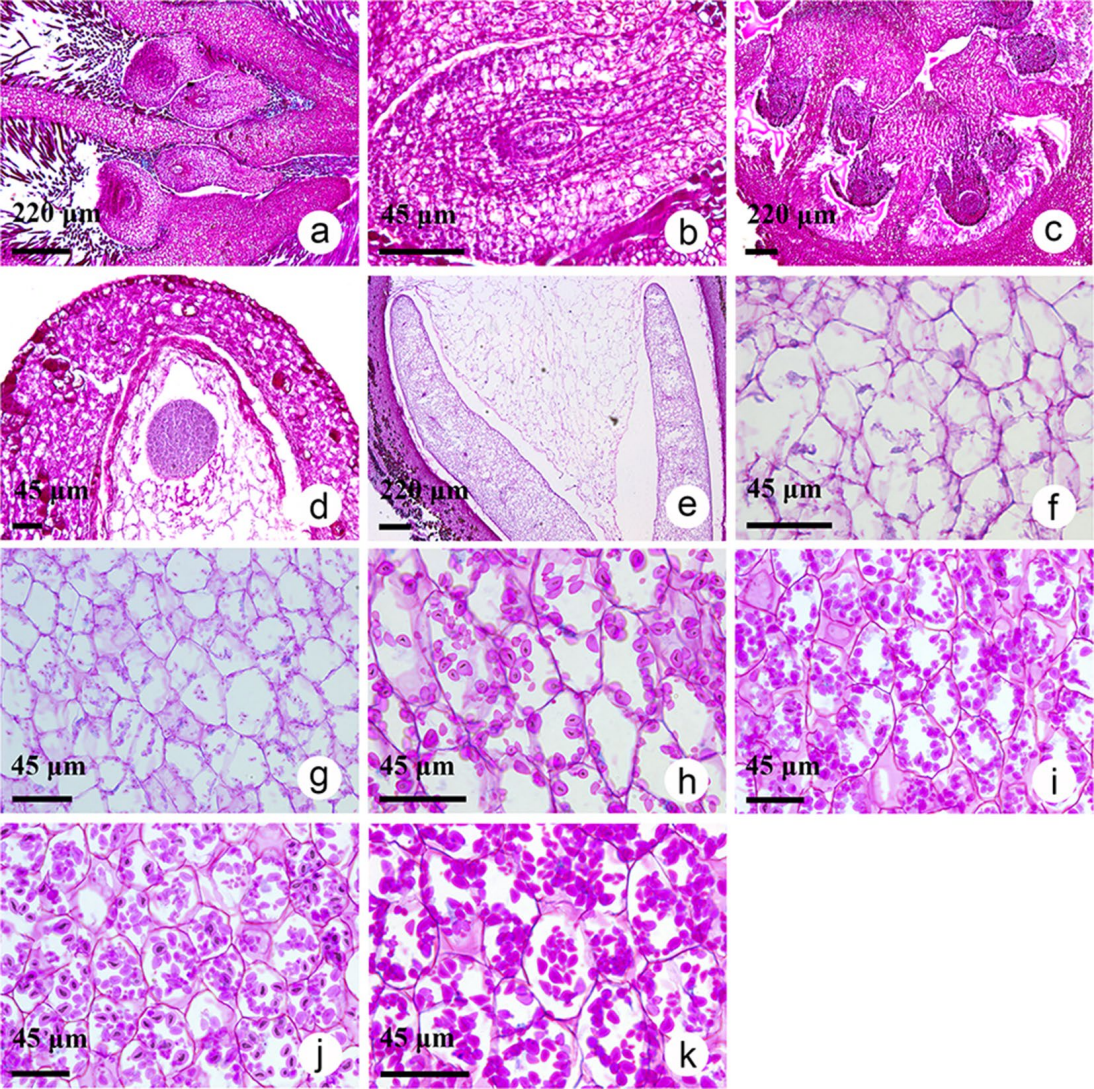
CCSK at various developmental stages stained with PAS. (**a**) Longitudinal section of ovary at 10 days after flowering (DAF). (**b**) Longitudinal section of ovule at 20 DAF. (**c**) Transverse section of ovary at 30 DAF. (**d**) Globular proembryo stage, 40 DAF. (**e**) Cotyledon at 50 DAF. (**f**) Cotyledon at 50 DAF. (**g**) Cotyledon at 60 DAF. (**h**) Cotyledon at 70 DAF. (**i**) Cotyledon at 80 DAF. (**j**) Cotyledon at 90 DAF. (**k**) Cotyledon at 100 DAF. Scale: **a**, **c**, **e** = 220 μm; **b**, **d**, **f**, **g**–**k** = 45 μm.

The characteristics of chestnut fruit growth and development described above prompted us to explore the dynamic accumulation patterns of starch granules. To this end, periodic acid-Schiff (PAS) staining was conducted on chestnut fruits at 10–40 DAF (removing the spiked involucres) and kernels (removing the spiked involucres, shells, and skin) at 50–100 DAF. Through microscopy, we found that the ovary of the fruit consisted of six to eight ventricles, each with two ovules (Fig. 2a, c), and that the specific developmental stage of fruit could not be determined (Fig. 2a–c) until the globular embryo stage (40 DAF) (Fig. 2d). Ten days later, two cotyledons began to grow, and no starch granules were found with further observation under a light microscope (Fig. 2e, f). Notably, few starch granules appeared until 60 DAF (Fig. 2g), after which a significant increase in starch granules was observed until the stage of full maturity (Fig. 2h–k), reflecting that the rapid accumulation period of starch occurred at 70–100 DAF, corresponded to the growth pattern of chestnut fresh weight (Fig. 1c). Also, it was found that starch granules of raw chestnuts were round and oval in shape, with a smooth external surface and eccentric hilum (Fig. 2h–k). Through microscopic observation of starch granules, we found that the chestnut starch granules accumulated continuously with fruit development, as was consistent with the dynamic changes of starch content, which may play a vital role in determining the critical accumulative period of chestnut starch. It was reported that common starch granules from different kinds of plants exhibited distinct morphologies ranging from round, truncated, lenticular, oval or polygonal^31–33^, of which the round and oval shape was identified in this work, in line with previous studies of the morphology of chestnut starch^7,34^.

### Dynamic changes in sucrose and starch contents in developing CCSK

**Figure 3:**
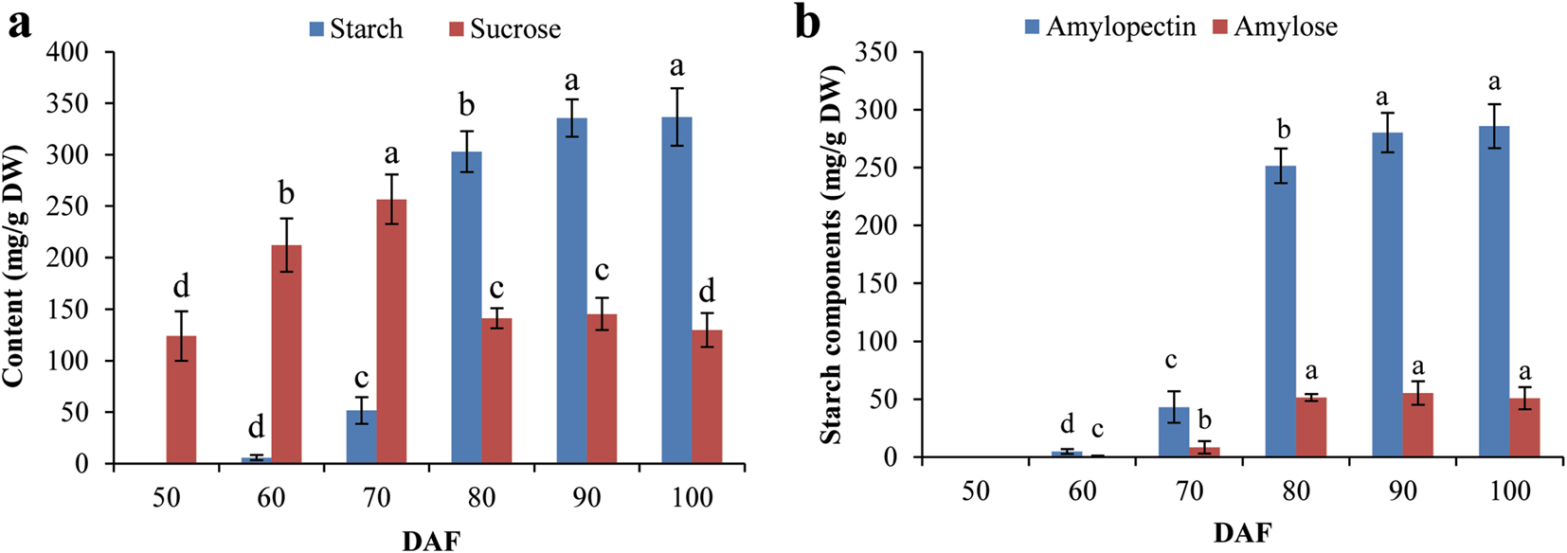
Sucrose and starch contents of CCSK at various developmental stages. (**a**) Sucrose and starch contents of CCSK at various developmental stages. (**b**) Amylose and amylopectin contents of CCSK at various developmental stages. Groups with distinct letters are significantly different at *p* < 0.05. Error bars display SD, n = 3.

Sucrose is the main sugar in Chestnut cultivars, eg. Aveleira, Judia, and Longal^35,36^ as well as the carbon and energy source from photosynthesis which are supplied to heterotrophic organs to initiate the biosynthesis of starch^37^. To explain the starch accumulation pattern and to address the relationship between sucrose content and starch accumulation, we analyzed dynamic changes in the sucrose and starch contents in developing CCSK at 50–100 DAF, and found that the starch content was about 60-fold higher at 90 DAF (335.60 ± 7.45 mg/g DW) than at 60 DAF (5.65 ± 0.24 mg/g DW), followed by stabilization at 100 DAF (Fig. 3a). These results revealed that active accumulation of starch occurs mainly during the middle-late stage (60–90 DAF), coinciding with fruit growth and starch granule accumulation patterns during CCSK development (Fig. 1c, Fig. 2). However, sucrose content increased significantly from 50 DAF (124.07 ± 1.25 mg/g DW) to 70 DAF (256.58 ± 3.52 mg/g DW), declined markedly at 80 DAF (141.36 ± 1.15 mg/g DW), and then remained stable at 80–100 DAF (Fig. 3a), implying that the majority of sucrose accumulation occurs before 70 DAF in developing CCSK. Hence, the rapid accumulation of sucrose appears to begin before major starch accumulation in developing CCSK, presumably to provide a carbon source (photosynthetic sucrose) for starch biosynthesis.

Notably, the ratio of amylopectin to amylose is a critical factor affecting starch structure, properties and functionalities of starch^38–41^. In order to promote the application of chestnut starch, it is crucial to analyze its component and accumulation pattern in the whole growth period. Here, using dual wavelength spectroscopy, we detected a significant difference in the contents of the two starch components (amylose and amylopectin) during CCSK development, of which amylopectin showed more abundant, and its content gradually increased to a peak at 100 DAF, a similar pattern was noted for amylose (Fig. 3b). Amylopectin accounted for more than 70.7% of the total starch content in fully mature CCSK (100 DAF) (Fig. 3b), comparable with previous studies in *C. crenata* (70.4%)^42^ and *C. sativa* (73.4%)^7^, but lower than that reported in *C. mollissima* (81.7%)^43^ and higher than another study in *C. sativa* (67.1%)^44^. These differences may be attributed to analysis of chestnuts with different geographic origins, or to different methods used for amylopectin determination and starch isolation^7^. In terms of amylopectin content of starch plants, Chinese chestnut fell in the range of normal level, compared with that low amylopectin starches were reported in corn varieties^45,46^, while wheat and potato were reported to be regular starches^31^. Owing to that amylose and amylopectin possess different properties and uses, the corresponding functions and products can be developed according to the content of starch components in chestnut fruit.

### Illumina sequencing and functional annotation in developing CCSK

To explore molecular regulatory mechanism of starch biosynthesis in developing CCSK, CCSK samples from six crucial periods (50, 60, 70, 80, 90, and 100 DAF) were selected for transcriptomic sequencing analysis based on the above results for fruit growth and starch granule accumulation as well as dynamic patterns of starch content during CCSK development (Figs. 1, 2, 3). A total of six cDNA libraries were constructed from these different developing CCSK, and then sequenced by Illumina HiSeq 2500 system. The raw data of transcriptome sequencing has been submitted to National Center for Biotechnology Information (The BioProject and biosample accession numbers are PRJNA578603 and SAMN1307483, respectively). An average of 42,446,929 clean reads was produced from six cDNA libraries after removing adaptor sequences and low-quality reads. All clean reads were mapped to the reference genome of Chinese chestnut using Tophat or bowtie2, resulting in a total of 38,146 unigenes with a mean length of 1164.19 bp (Additional file 1: Table S1 and Additional file 2: Table S2), which is longer than those from Siberian apricot (829.62 bp)^29^, *Centella asiatica* (474 bp)^47^, and Yellow Horn (462 bp)^48^. These results implied that our Illumina sequencing successfully captured most expressed genes, ensuring the reliability of the transcriptome data.

To better identify unigenes to be involved specifically in starch biosynthesis in developing CCSK, all obtained unigenes were functionally annotated using the BLASTX algorithm in public databases with an E-value < 10^−5^. Of 38,146 unigenes, 36,284 (95.12%), 29,424 (77.14%), 21,244 (55.69%), 12,091 (31.70%), and 8345 (21.88%) unigenes in developing CCSK showed high similarity to known proteins in the National Center for Biotechnology Information nonredundant (NCBI Nr), Swiss protein (SwissProt), eukaryotic orthologous groups (KOG), Gene Ontology (GO) and Kyoto Encyclopedia of Genes and Genomes (KEGG) databases, respectively (Additional file 3: Table S3). Of these, 21,477 (56.30%) were annotated in at least one database. To further explore the interactions of all annotated unigenes, we preformed GO, KOG functional enrichment and KEGG pathway analyses. These analyses resulted in 12,091 (31.70%) unigenes assigned into three main GO categories and 64 subcategories, 21,244 (55.69%) unigenes into 25 KOG classifications (Additional file 4: Fig. S1), and 8345 (21.88%) unigenes classified into 392 KEGG pathways and 981 types of enzymes.

**Figure 4:**
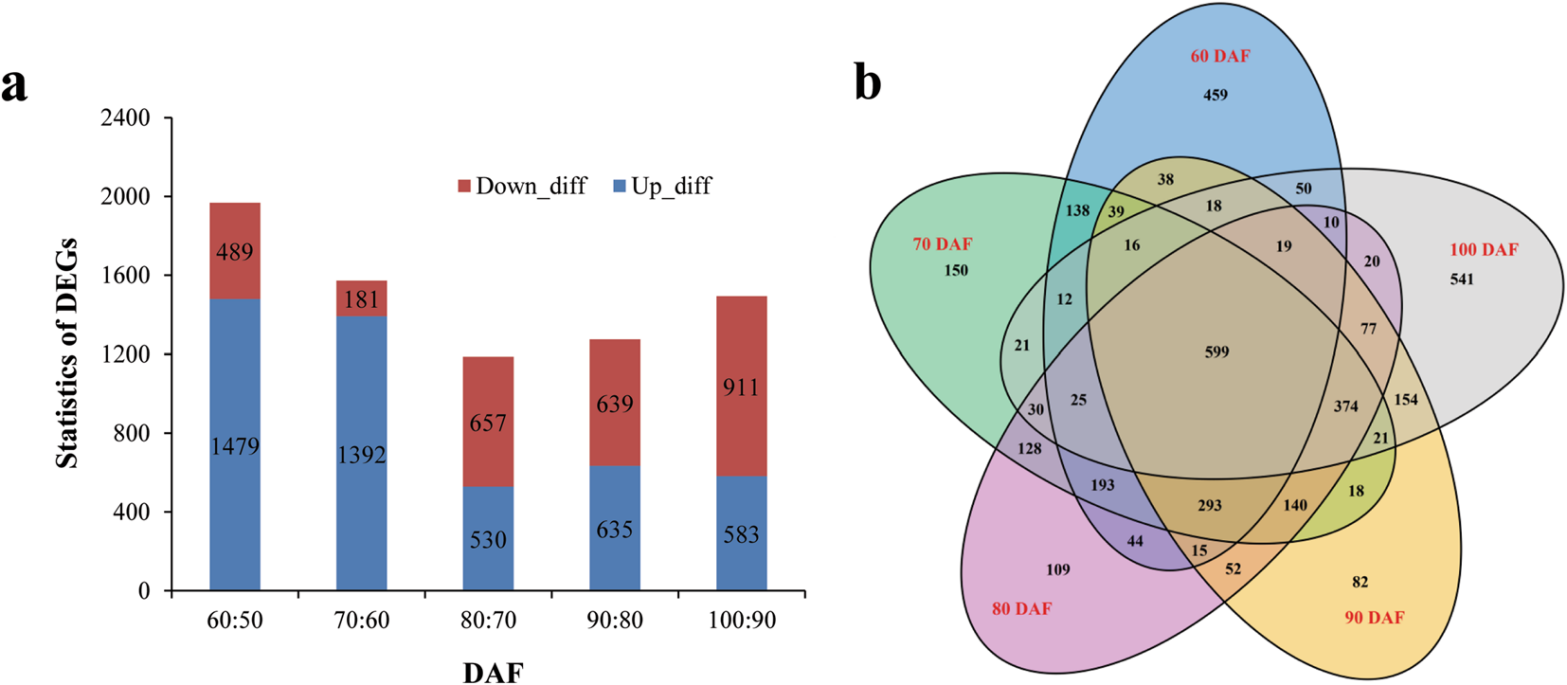
Number and distribution of differentially expressed genes in developing CCSK. (**a**) Numbers of upregulated and downregulated transcripts. (**b**) Numbers of differentially expressed genes.

To identify the differentially expressed genes (DEGs) in developing CCSK, the above obtained unigenes were screened by fragments per kilobase per million reads (FPKM) values using the DESeq method (Additional file 5: Fig. S2). It was found that 60:50 DAF showed the most upregulated (1479), and 80:70 had the fewest upregulated unigenes (530). However, 100:90 DAF had the most downregulated (911), and 70:60 the fewest ones (181) (Fig. 4a). By the Venn diagram analysis, a total of 3885 unigenes were characterized to be expressed during the whole development of CCSK, whereas 459, 150, 109, 82, and 541 unigenes were expressed specifically at 60, 70, 80, 90, and 100 DAF, respectively (Fig. 4b). Moreover, we performed the GO function enrichment and KEGG pathway analysis of DEGs in developing CCSK, and a total of 130 DEGs related to “Starch and sucrose metabolism” were identified by KEGG pathway analysis, with 70:60 DAF exhibiting the most upregulated (21) and 60:50 DAF the most downregulated (17) DEGs (Additional file 6: Fig. S3 and Additional file 7: Fig. S4). These results indicated that transcripts of many CCSK unigenes may specifically respond to different developing stages, most of which are likely involved in CCSK development and starch biosynthesis.

Altogether, the present results reflected the reliability of our transcriptome sequencing analysis, and a total of 239 unigenes were screened to be potentially involved in starch accumulation during CCSK development by KEGG enrichment annotation, among which 238 unigenes were further validated by Swissprot annotation, 136 unigenes by KOG and 117 unigenes by GO enrichment analysis (Additional file 8: Table S4). Most of them were related to sucrose metabolism, glycolysis, metabolite transport, starch biosynthesis or transcriptional regulation, indicating a complex mechanism of transcriptional regulation driving starch biosynthesis and accumulation during CCSK development.

### Sucrose cleavage in the cytosol of developing CCSK

**Figure 5:**
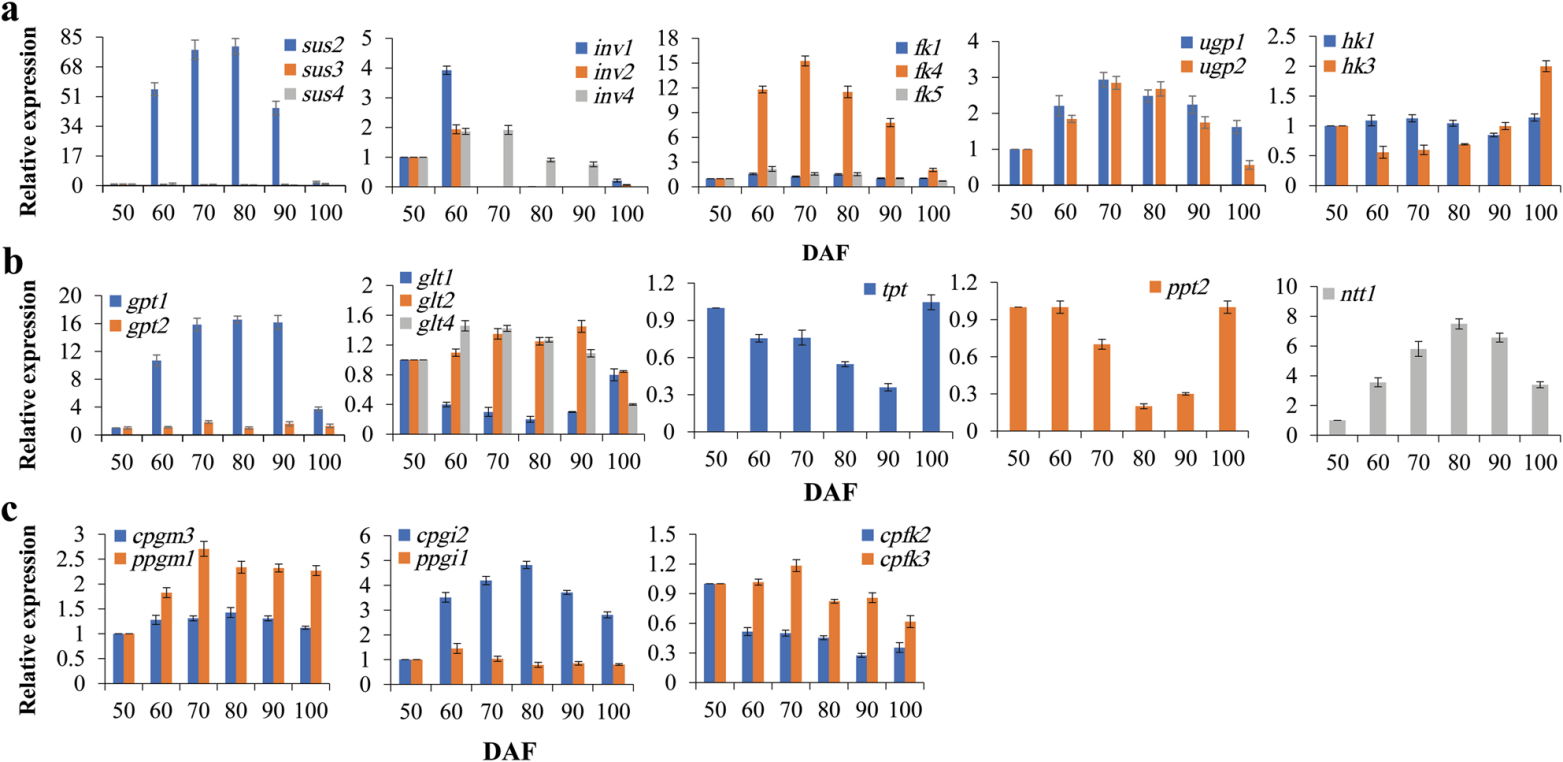
Transcriptional expression analysis of genes encoding enzymes involved in sucrose cleavage and carbon allocation in developing CCSK via qRT-PCR. Error bars display SD, n = 3. (**a**) Differential transcription patterns of enzymes related to sucrose cleavage determined via qRT-PCR. (**b**) Differential transcription patterns of transporters related to carbon allocation determined via qRT-PCR. (**c**) Differential transcription patterns of enzymes related to carbon allocation determined via qRT-PCR. *cpgm3*, cytosolic *pgm3*; *ppgm1*, plastidial *pgm1*; *cpgi2*, cytosolic *pgi2*; *ppgi1*, plastidial *pgi1*; *cpfk*, cytosolic *pfk*.

In heterotrophic organs, starch biosynthesis is initiated by the supply of sucrose as a source of carbon and energy from photosynthetic tissues, and channeling of incoming sucrose for metabolism requires its cleavage by sucrose synthase (SUS) or invertase (INV)^37,49,50^. In general, SUS reversibly catalyzes sucrose and uridine diphosphate (UDP) to fructose (Fru) and UDP-glucose (UDPG) in the cytoplasm of the storage organ, whereas INV catalyzes the irreversible hydrolysis of sucrose to glucose (Glu) and Fru^51–54^. Thus, to fully understand the allocation of available carbon from sucrose to starch synthesis in the developing CCSK, it was essential to concretely analyze the differential transcription profiles of genes for sucrose-cleaving enzymes in developing CCSK. Through sequence analysis, we identified three cytosolic SUS isoforms (SUS2/3/4) and three INV isoforms (cell wall INV1/4 and vacuolar INV2) with differential transcription in developing CCSK, of which only SUS2 displayed high transcription levels at 50–100 DAF, whereas INV1 and INV4 showed upregulated transcripts at 50–60 DAF and 50–70 DAF, respectively (Additional file 9: Table S5), in accordance with our qRT-PCR results (Fig. 5a). Our findings that the transcript level of SUS2 was higher than that of INV1/4 during CCSK development and that SUS2 transcripts increased notably during the active starch accumulation period in developing CCSK (Figs. 3a and 5a) revealed that SUS2 may be the main enzyme responsible for initial sucrose cleavage in developing CCSK. Supporting this conclusion, differential transcripts were detected for cytosolic fructokinase (FK1/4/5) (upregulation for FK4 and upregulation for FK1/5 at 50–80DAF) and hexokinase (HK1/3) (downregulation or nearly stable at 50–90 DAF), and high transcript levels were also noted for cytosolic UDPG pyrophosphorylase (UGP1/2), which is involved in the conversion of UDPG to glucose-1-P (G1P) (Additional file 9: Table S5 and Fig. 5a). These results indicated that strong coordination of transcription among SUS2, FK1/5, and UGP1/2 may contribute to generation of cytosolic hexose phosphate (G1P and F6P) pool in developing CCSK, in accordance with previous studies of developing kiwifruit and oilseed plants *Ricinus communis*, *Brassica napus*, *Euonymus alatus*, and *Tropaeolum majus*^55,56^. This finding led us to hypothesize that carbon is supplied to the developing CCSK primarily as sucrose, as reported in developing peach fruits^57^. Sucrose-cleaving enzymes are crucial to the development, growth, and carbon partitioning of plants^50^, but the sucrose cleavage activity of SUS is linked to starch biosynthesis, providing substrates for starch accumulation in sink organs^58^, suggesting that SUS2 may provide a carbon source for starch synthesis in developing CCSK by cleaving sucrose. In addition to SUS, we found abundant transcripts of cell-wall INV1/4 at 50–70 DAF, and a similar trend in fruit growth (Figs. 1c and 5a), indicating that these enzymes may also contribute to sucrose metabolism in developing CCSK, as also noted in the fruits of tomato and *Lindera glauca*^59,60^. Co-regulation of SUS2 and INV1/4 activities may allow specific response to carbon allocation, driving the growth, development, and starch accumulation of developing CCSK. Overall, sucrose in the cytosol was cleaved mainly by SUS2 into UDPG and Fru, from which G1P and F6P were produced by UGP and FK, creating a hexose phosphate pool as a carbon source in the developing CCSK.

### Source of G1P for starch accumulation in the amyloplast of developing CCSK

G1P is known to act as a glucosyl donor or substrate for the biosynthesis of ADPG, a critical precursor for starch biosynthesis^61^. Therefore, it was necessary to determine the potential source of G1P for starch biosynthesis in our experiments. G1P, which is mainly derived from cytosolic G6P in developing seeds of plants, is transported by glucose 6-phosphate (G6P) translocator (GPT) to the plastid, where it is converted to G1P by plastidial phosphoglucomutase (PGM)^62,63^. Meanwhile, both PGM and glucose-6-phosphate isomerase (PGI) have been shown to have roles in the interconversion of hexose phosphate (G1P, F6P, and G6P)^64^.

In this study, through a combination of sequencing and qRT-PCR analyses, we identified the differential transcript levels of cytosolic PGI2 and PGM3, as well as plastidial PGI1 and PGM1, in developing CCSK (Additional file 9: Table S5 and Fig. 5c). We found that the cytosolic PGI2 transcript level was upregulated during CCSK development, whereas plastidial PGI1 transcripts increased only at 50–60 DAF, and both cytosolic PGM3 and plastidial PGM1 were upregulated in developing CCSK. Given that enzymes (SUS2, FK1/5, and UGP1/2) related to the production of G1P and F6P were highly expressed during CCSK development (Fig. 5a), the transcript abundance of both cytosolic PGI2 and PGM3 may play pivotal roles in the conversion of sucrose cleavage products (G1P and F6P) into G6P to support further starch synthesis in the amyloplast, as observed in potato tuber and the biopolymer gellan gum^64–66^. This finding prompted us to explore interchange of glycolytic intermediates between the cytosol and plastid in developing CCSK. Intriguingly, differentially expressed genes encoding a glucose transporter (GLT1/2/4), G6P transporter (GPT1/2), triose phosphate transporter (TPT), and phosphoenolpyruvate (PEP) transporter (PPT2) were annotated in the developing CCSK (Additional file 9: Table S5). Among these genes, GPT1 showed the greatest transcript abundance and GLT4 was also upregulated at 50–90 DAF, whereas GPT2 showed an up-down-up expression trend and a stable low level or downregulation was observed for TPT and PPT in developing CCSK (Fig. 5b). Notably, the transcript level of GPT1 was much higher (about 6.8-fold and 5.7-fold, respectively) than those of GPT2 and GLT4, highlighting the importance of GPT1 for G6P transport into the plastid from the cytosol during CCSK development, as documented in *Vicia* seeds^67^. This conclusion was supported by the finding that the transcript abundance of GPT (significant upregulation) was much higher than that of PFK (downregulation), which catalyzes phosphorylation of F6P to fructose-1,6-bisphosphate (F1,6P), a key regulatory step in glycolytic pathway. Upregulation was observed for cytosolic PGI2 transcripts, an enzyme that converts F1P to G6P (Fig. 5b, c). In addition, adenylate/nucleotide translocator (NTT) was identified as having highly significantly upregulated expression across the plastid membrane of CCSK, which is consistent with dynamic accumulation of starch, indicating that GPT and NTT together may provide carbon skeletons and ATP for starch synthesis in developing CCSK. This possibility was supported by research using transgenic potato plants, in which GPT and NTT co-limit starch content and yield^68^.

Given high transcriptional pattern for plastidial PGM1 noted above, we speculated that G6P imported into the amyloplast was converted via PGM1 to G1P, which can be metabolized to starch, as noted in a study on the potato tuber^69^. This conclusion was confirmed by our finding that transcript of plastidial PGI was upregulated only at 50–60 DAF and downregulated thereafter, corresponding to the upregulation of plastidial PGM1 in the developing CCSK.

### Transcript analysis of starch biosynthesis-related genes in developing CCSK

**Figure 6:**
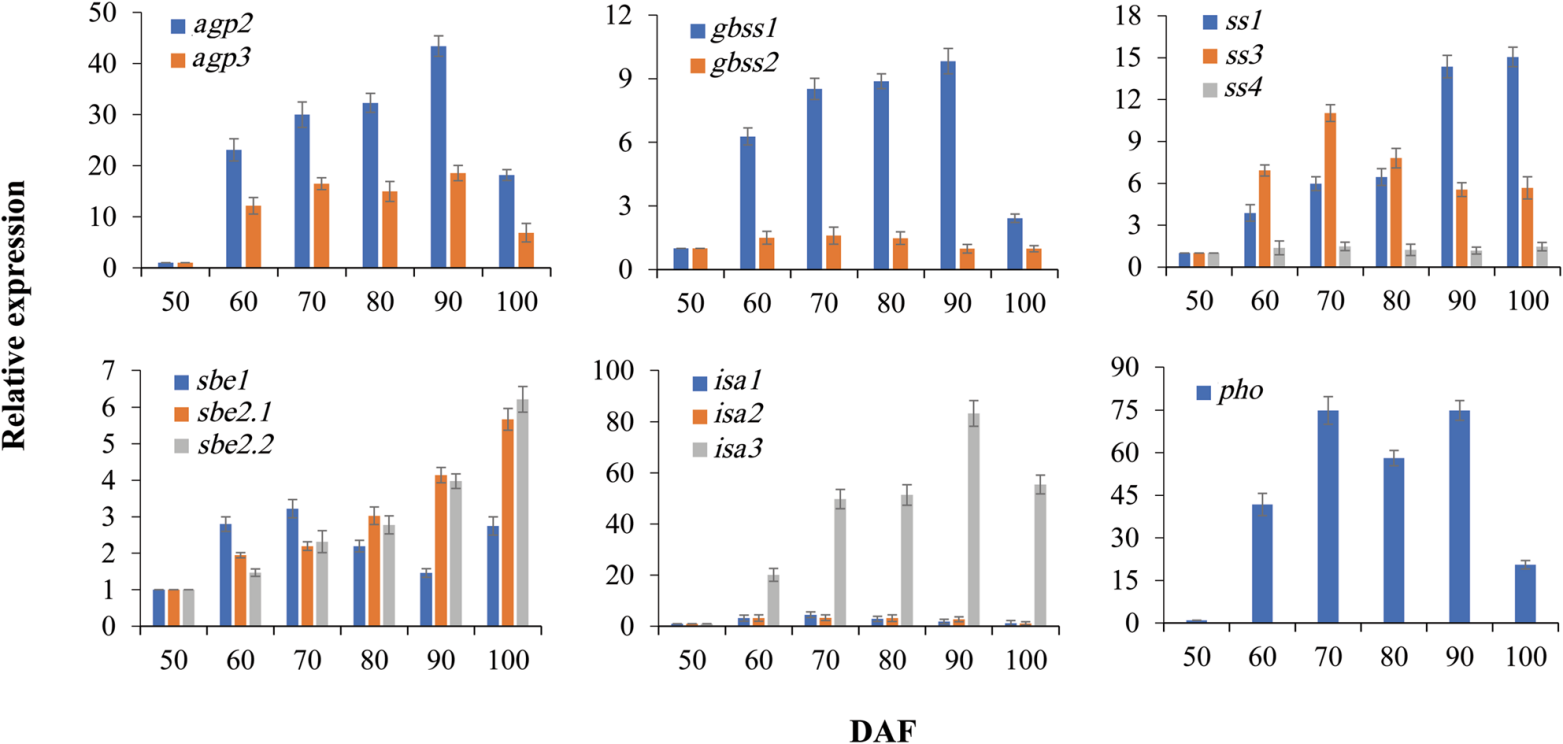
Transcriptional expression analysis of genes encoding enzymes involved in starch biosynthesis in developing CCSK via qRT-PCR. Error bars display SD, n = 3.

It was suggested that starch in heterotrophic organs is synthesized primarily in the amyloplast through the coordinated action of multiple enzymes, including ADP-glucose pyrophosphorylase (AGPase), granule-bound starch synthase (GBSS), starch synthase (SS), starch branching enzymes (SBE), isoamylase-type starch-debranching enzyme (ISA), and phosphorylase (PHO)^13,70,71^. In this study, the genes encoding all of these enzymes related to starch biosynthesis in the amyloplast of developing CCSK were characterized by Illumina sequencing analysis (Additional file 9: Table S5). To clarify the potential contributions of AGPase, GBSS, SS, SBE, ISA, and PHO to starch synthesis in developing CCSK, their temporal transcript patterns were determined using qRT-PCR at various developmental stages. We found that transcripts of AGP2/3, GBSS1, SS1/3, SBE2.1/2.2, ISA3, and PHO all were significantly upregulated at 50–90 DAF, whereas those of SBE1, GBSS2, and ISA1/2 had high transcript abundance at early developmental stages (50–70 DAF), and low transcript levels were observed for AGP1 and SS4 throughout development (Additional file 9: Table S5 and Fig. 6). These results indicated that AGP2/3, GBSS1, SS1/3, SBE2.1/2.2, ISA3, and PHO may play major roles in the starch synthesis pathway in developing CCSK.

ADPG is a direct precursor essential to starch biosynthesis, and therefore it is particularly important to determine the mechanism of ADPG generation for starch synthesis in developing CCSK. Studies have shown that AGPase is involved in sucrose-to-starch conversion, primarily through promoting the influx of photosynthetic carbon and the resulting ADPG needed as a sugar donor to produce starch^13,62,63,72–74^, which is supported by our finding that AGP2/3 exhibited a highly correlated temporal pattern with active starch accumulation in CCSK at 50–90 DAF (Figs. 3a and 6). This result highlights the role of AGP2/3 in starch biosynthesis during CCSK development, similar to previous studies in sweet potato and transgenic maize^70,75^.

As the two main components of starch, amylose and amylopectin are synthesized by GBSS and SS, respectively^70^. In developing CCSK, a total of five isoforms were identified (GBSS1/2, SS1/3/4), among which GBSS1 and SS1 exhibited markedly upregulated expression at 50–90 DAF and SS3 showed upregulated expression at 50–70 DAF, whereas GBSS2 and SS4 showed low and stable expression, in accordance with the results of qRT-PCR analysis (Fig. 6). Moreover, we found that the transcript expression levels of GBSS1 and SS1 corresponded to the accumulation of amylose and amylopectin, respectively, in CCSK at 50–90 DAF (Fig. 3b). Thus, the data clearly revealed that GBSS1 and SS1 play the major roles in the biosynthesis pathway of amylose and amylopectin, respectively, in developing CCSK, as reported previously in *Arabidopsis* leaves and potato tubers^76,77^.

Both SS and SBE are known to play central regulatory role in the biosynthesis of starch^38,78^. For amylopectin production, aside from SS, three other enzymes also play important role in forming the polymer structure, in which SBE cleaves the α-1,4 linkage of starch and transfers the chain onto a glucan chain with α-1,6 bond, altering starch structure; ISA hydrolyzes some branches, tailoring the polymer structure crucial for production of crystalline starch granules^14,79^; and PHO plays a notable role in the modification of amylopectin structure^71^. Hence, we investigated the potential roles of these enzymes in amylopectin biosynthesis during CCSK development. Three isoforms of SBE (SBE1/2.1/2.2) and ISA (ISA1/2/3), as well as PHO, were identified in developing CCSK, and importantly, transcript abundance of SBE2.1/2.2, ISA1/2/3 and PHO was found to be highly correlated with the accumulation of amylopectin (Figs. 3b and 6), indicating that they may make major contribution to amylopectin biosynthesis in developing CCSK, as also reported for potato tubers^80,81^. Moreover, transcripts of both G6P transporters (GPT1/2) increased over the active starch synthesis period (Fig. 5b), revealing that import of G6P from the cytosol to amyloplasts was critical for starch synthesis in developing CCSK. Meanwhile, upregulated expression of PGM1, AGP2/3, GBSS1, SS1/3, SBE2.1/2.2, ISA1/2/3, and PHO ensured that this imported carbon was used for starch synthesis.

Also noteworthy was that both ISA1 and ISA2 were upregulated at 50–70 DAF, while ISA3 was significantly upregulated at 50–90 DAF, and its transcript level was about 3–5 times higher than that showed by ISA1/2, which was in line with the dynamic changes in amylopectin content of developing CCSK (Figs. 3b and 6), indicating a paramount role of ISA3 for amylopectin biosynthesis. This was in contradiction with the result that *ISA3* mutant could result in starch breakdown of *Arabidopsis*^82^. It has been shown that ISA plays a key role in the initiation of starch granule formation in barley mutant and transgenic rice^83,84^. Given our results of starch granule accumulation and transcripts of ISA1/2/3 in the early stage (50–70 DAF) of CCSK development (Figs. 2e–h and 6), it seems certain that ISA may play a critical role for starch granule initiation in developing CCSK.

**Figure 7:**
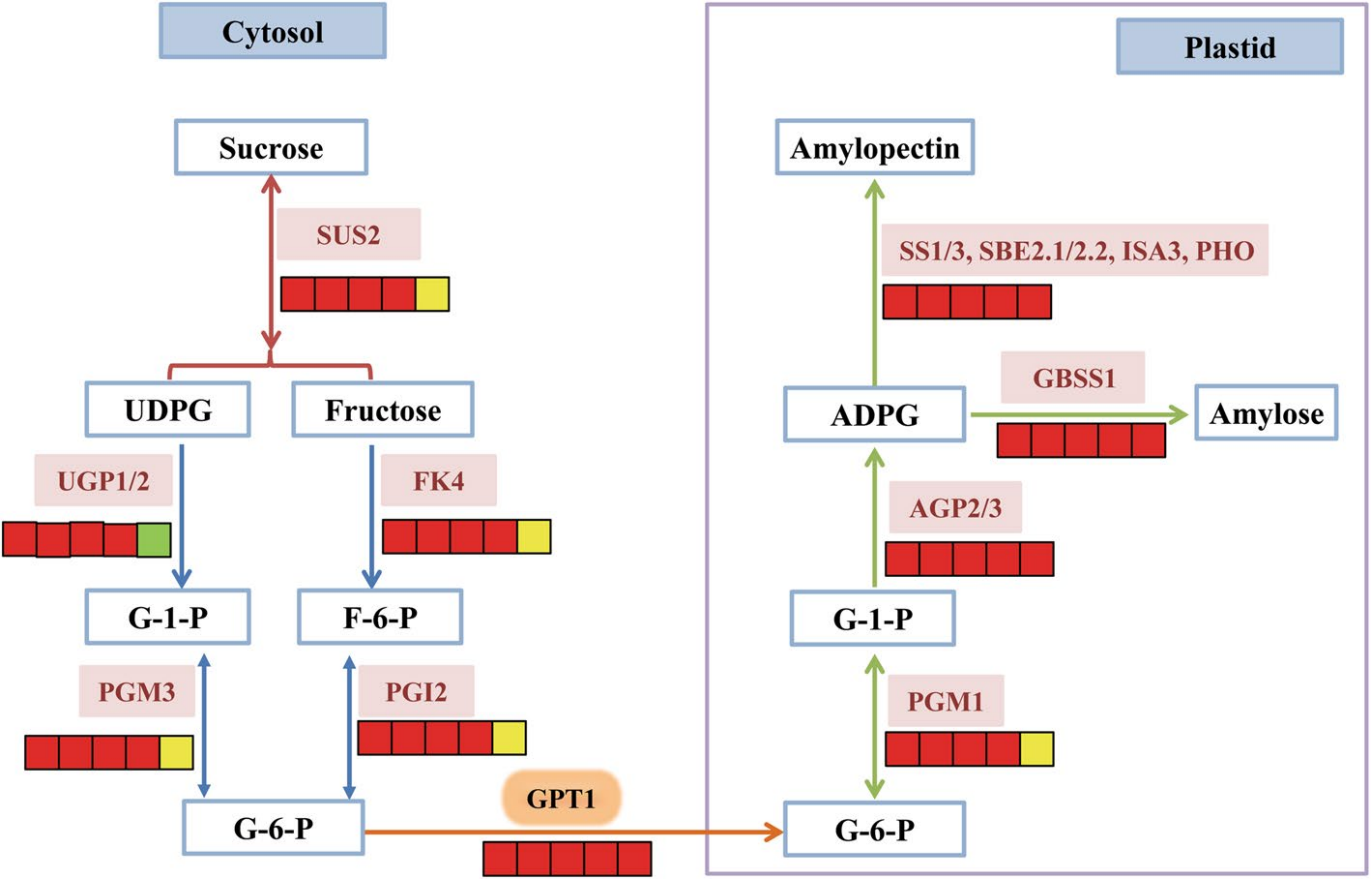
Temporal expression patterns of enzymes involved in starch biosynthesis in developing CCSK. The icons below each enzyme show the result of DESeq analysis, from left to right: 60:50 DAF, 70:60 DAF, 80:70 DAF, 90:80 DAF and 100:90 DAF; red, up-regulation; yellow, no significant difference; green, down-regulation.

In summary, sucrose pumped into the cytoplasm was cleaved and carbon-distributed to form G6P, which entered the amyloplast through GPT, generated G1P through pPGM, and eventually produced starch via the coordinated actions of multiple enzymes in CCSK (Fig. 7).

## Conclusion

The starchy Chinese chestnut is a potential substrate for fermentation and holds promise for development as industrial raw materials or biological products. To fully understand the temporal patterns of starch accumulation, ten samples collected throughout the developmental period of CCSK were analyzed. Based on starch and sucrose content measurement and microscopic observation of CCSK at various developmental stages, six experimental samples from crucial periods (50, 60, 70, 80, 90, and 100 DAF) were selected for comparative deep transcriptomic analysis. The resulting 38,146 unigenes with a mean length of 1164.19 bp were obtained and deposited in public database, massively enriching the dataset available for Chinese chestnut. By using the DESeq method, a total of 1968, 1573, 1187, 1274, and 1494 differentially expressed unigenes were identified specifically at 60:50, 70:60, 80:70, 90:80, and 100:90 DAF, respectively, of which 239 unigenes were characterized to be potentially involved in starch accumulation in developing CCSK. Notably, the application of an integrated analysis of comparative transcriptome sequencing and temporal pattern of starch accumulation has led to the identification of some crucial regulatory enzymes (SUSY2, PGM, PGI, GPT, NTT, AGP3, AGP2, GBSS1, SS1, SBE1, SBE2.1, SBE2.2, ISA1, ISA2, ISA3, and PHO) responsible for the biosynthesis of G-1-P, ADPG, and starch of developing CCSK. Also, the dynamic transcript patterns of some key related genes (*susy2*, *pgi2*, *gpt1*, *agp2*, *agp3*, *gbss1*, *ss1, sbe1, sbe2.1, sbe2.2, isa1, isa2, isa3*, *and pho*) in developing CCSK were validated by qRT-PCR detection. Together, our findings will provide a better insight into regulatory mechanism of high starch production and may also provide both a rich source of data and of considerable interest to those studying starch plants.

## Methods

### Plant materials

Developing fruits from *Castanea mollissima* Blume cv. Huaifeng were obtained from 10-year-old plus tree located in Jiufeng National Forest Park (E 116° 28′, N 39° 54′) of Beijing, China. The developmental stages of fruits from flowering to seed maturity were observed from June to September. Flowers that were at the anthesis stage simultaneously were marked, and fruits were harvested at 10, 20, 30, 40, 50, 60, 70, 80, 90, and 100 DAF. Fifteen fruits from the randomly selected 3 trees (5 fruits each tree) of each developing stage were collected each time, and then immediately frozen in liquid nitrogen and stored at − 80 °C until use.

### Fruit development and dynamic accumulation of starch in developing Chinese chestnut fruits

The characteristics of chestnut fruits were observed throughout fruit growth (10–100 DAF). Fruit development was expressed using the fruit shape index (length/diameter) and fresh weight at different developmental stages. To investigate the accumulation pattern of starch, we examined dynamic changes of starch granules in the fruits. Fresh fruits were collected from the tree, fixed in formalin-acetic acid-alcohol (FAA) solution and stored in 70% ethanol at 4 °C until use. Then, samples were prepared as 8-μm sections using the conventional paraffin section method^85^, stained using the PAS method, and finally observed and photographed using a Leica DM 6000 light microscope.

After the kernels (50–100 DAF) were dried in a 45 °C oven, each dried sample was ground into fine powder with a mortar and pestle, followed by filtration through a 30-mesh sieve prior to use. The sucrose content (about 50 mg of sample powder) was determined using a previously described method^86^. The contents of starch components (amylose and amylopectin, about 50 mg of sample powder) were determined through dual wavelength spectroscopy, with amylopectin measured at 550 nm and 695 nm and amylose measured at 617 nm and 475 nm, using a spectrophotometer (UV-2102C)^87^. All the determinations were performed in triplicate.

### cDNA library construction, sequence analysis and alignment

Based on microscopic inspection of starch grain accumulation and analysis of starch content in developing CCSK, samples from six crucial stages (50, 60, 70, 80, 90, and 100 DAF) were selected for transcriptomic analysis. Two fresh seeds of each fruit (3 samples per developing period) were cut into small pieces and mixed together, and then the equal weights of three biological duplicates from each developmental stage were fully mixed for total RNA extraction using the RNA Isolation Kit following the manufacturer’s protocol. Purified RNA was quality-tested and quantified using a Nanodrop ND-1000 spectrophotometer (Wilmington, DE, USA), and all samples showed 260/280 nm ratios of 2.1–2.2. cDNA library construction and normalization were performed as described previously^29^. Illumina sequencing was conducted on the HiSeq 2500 sequencing system. After removal of reads containing poly-N and low quality reads, the remaining clean reads were mapped to the reference *C. mollissima* genome using Tophat or bowtie2^88,89^ (http://tophat.cbcb.umd.edu/), from which unigenes were obtained.

### Differential expression analysis of unigenes and sequence annotation

Unigene expression levels were calculated as FPKM using the Cufflinks program^90^, and read counts for each gene were obtained using htseq-count^91^. The levels of gene expression in various samples were compared using the DESeq method^92^, with *P*-value < 0.05 and fold-change > 2 or fold-change < 0.5 used as thresholds indicating significant differences in gene expression. Databases including the NCBI Nr (https://www.ncbi.nlm.nih.gov/), SwissProt^93^, and KOG^94^ were used to annotate the unigenes with an E-value cut-off of 10^−5^. GO^95^ and KEGG^96,97^ pathway enrichment analyses of DEGs were both performed using R, based on a hypergeometric distribution^48^.

### *Transcription expression analysis *via* qRT-PCR*

Total RNA was extracted as described for cDNA library preparation and was reverse transcribed using HiScript II QRT SuperMix for qPCR (+ gDNA wiper) (Vazyme, R223-01). The primers were designed using Roche LCPDS2 software based on mRNA sequences obtained from the NCBI database (Additional file 10: Table S6), and 18S rRNA was used as the reference gene. The qRT-PCR was performed with three replicates of each reaction using QuantiFast SYBR Green PCR Kit (Qiagen, Hilden, Germany) following the manufacturer’s specifications. The expression levels of genes were calculated using the 2^−ΔΔCt^ method^98^.

### Statistical analysis

The data (fruit weight, fruit shape index, the contents of sucrose, starch, amylopectin and amylose) reported in the figures are averages of at least three different determination. Microsoft Excel Statistical Software (Microsoft Office Excel 2016) software was used for statistical analysis. The level of significance used for all the statistical tests was 95%.

## Supplementary Information

Supplementary Legend.

Supplementary Table S1.

Supplementary Table S2.

Supplementary Table S3.

Supplementary Figure S1.

Supplementary Figure S2.

Supplementary Figure S3.

Supplementary Figure S4.

Supplementary Table S4.

Supplementary Table S5.

Supplementary Table S6.

## Abbreviations

CCSK: Chinese chestnut seed kernels
DAF: Days after flowering
RNA-Seq: RNA sequencing
qRT-PCR: Quantitative real-time PCR
DEGs: Differentially expressed genes
SUS: Sucrose synthase
INV: Invertase
FK: Fructose kinase
UGP: Uridyl diphosphate glucose pyrophosphorylase
HK: Hexokinase
GPT: Glucose-6-phosphate/phosphate transporter
GLT: Glucose transporter
TPT: Triose phosphate/phosphate translocator
NTT: ATP/ADP-transporter
PPT: Phosphoenolpyruvate/phosphatetranslocator
PGM: Phosphoglucomutase
PGI: Glucose-6-phosphate isomerase
PFK: 6-Phosphofructokinase
AGP: Adenosine diphosphate-glucose pyrophosphorylase
GBSS: Granule-bound starch synthase
SS: Starch synthase
SBE: 1,4-α-Glucan branching enzyme
ISA: Isoamylase type starch debranching enzyme
PHO: Phosphorylase
UDPG: Uridine diphosphate glucose
G-1-P: Glucose-1-phosphate
F-6-P: Fructose-6-phosphate
PGM: Phosphoglucomutase
G-6-P: Glucose-6-phosphate
