# Supplementary material for: Transcriptome analysis of genes involved in starch biosynthesis in developing Chinese chestnut (Castanea mollissima Blume) seed kernels
Source: Sci Rep. 2021 Feb 11;11:3570. doi: 10.1038/s41598-021-82130-6 (PMC7878784; doi:10.1038/s41598-021-82130-6)
Supplement: Supplementary file 1 — Supplementary Legend. [file 41598_2021_82130_MOESM1_ESM.pdf]

Transcriptome analysis of genes involved in starch biosynthesis in developing Chinese chestnut (*Castanea mollissima* Blume) seed kernels

Lingling Shi, Jia Wang, Yujun Liu, Chao Ma, Sujuan Guo, Shanzhi Lin*, Jianzhong Wang*

Beijing Advanced Innovation Center for Tree Breeding by Molecular Design, College of Biological Sciences and Biotechnology, National Engineering Laboratory for Tree Breeding, Beijing Forestry University, Beijing 100083, China

* Correspondence: Corresponding author: Tel/Fax +86-10-62336114; szlin@bjfu.edu.cn

Descriptive captions for supplementary files

Additional file 1: **Table S1.** Raw data and valid data statistics. (XLSX 11 kb)

Additional file 2: **Table S2.** Summary of CCSK sequencing and alignment. (XLSX 10 kb)

Additional file 3: **Table S3**. Functional annotation statistics of of unigenes in developing CCSK. (XLSX 10 kb)

Additional file 4: **Fig. S1.** Functional annotation of unigenes in developing CCSK (TIFF 295 kb) (a) Histogram presenting Gene Ontology classification. The results are summarized in three main categories: biological processes, cellular components, and molecular functions. The x-axis indicates GO categories and the y-axis indicates the number of genes. (b) Histogram presentation of KOG classification. A total of 21,244 unigenes were assigned to 25 classifications. Capital letters on the x-axis indicate the KOG categories, listed to the right of the histogram, and the y-axis indicates the number of unigenes.

Additional file 5: **Fig. S2** Comparative unigene expression profiles in developing CCSK. (TIFF 762 kb)

Additional file 6: **Fig. S3** Go enrichment of DEGs in developing CCSK (TIFF 18,949 kb)

Additional file 7: **Fig. S4** KEGG enrichment of DEGs in developing CCSK (TIFF 6262 kb)

Additional file 8: **Table S4.** Unigenes potentially involved in starch accumulation in developing CCSK (XLSX 70 kb)

Additional file 9: **Table S5.** Detailed information about annotated genes involved in starch accumulation in developing CCSK. (XLSX 531 kb)

Additional file 10: **Table S6.** Information about selected genes and primers used for qRT-PCR analysis in this study. (XLSX 11 kb)
