# Supplementary figures and images for: Transcriptome analysis of genes involved in starch biosynthesis in developing Chinese chestnut (Castanea mollissima Blume) seed kernels
Source: Sci Rep. 2021 Feb 11;11:3570. doi: 10.1038/s41598-021-82130-6 (PMC7878784; doi:10.1038/s41598-021-82130-6)

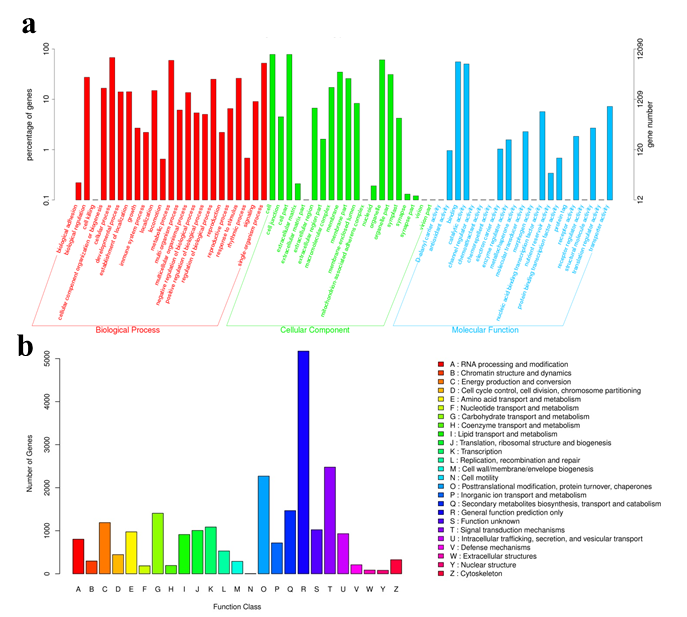

Supplement: Supplementary file 5 — Supplementary Figure S1. [file 41598_2021_82130_MOESM5_ESM.tif]

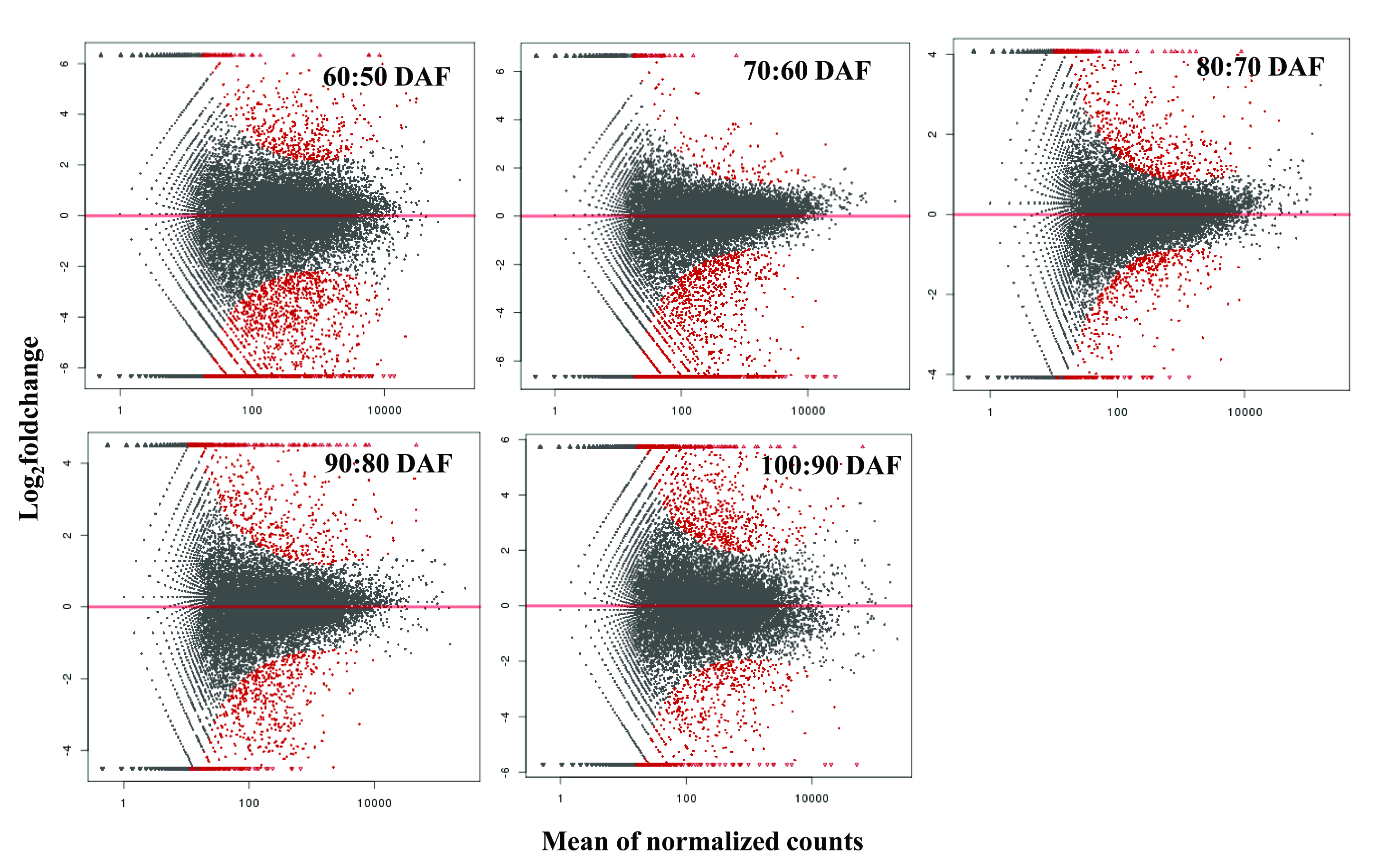

Supplement: Supplementary file 6 — Supplementary Figure S2. [file 41598_2021_82130_MOESM6_ESM.tif]

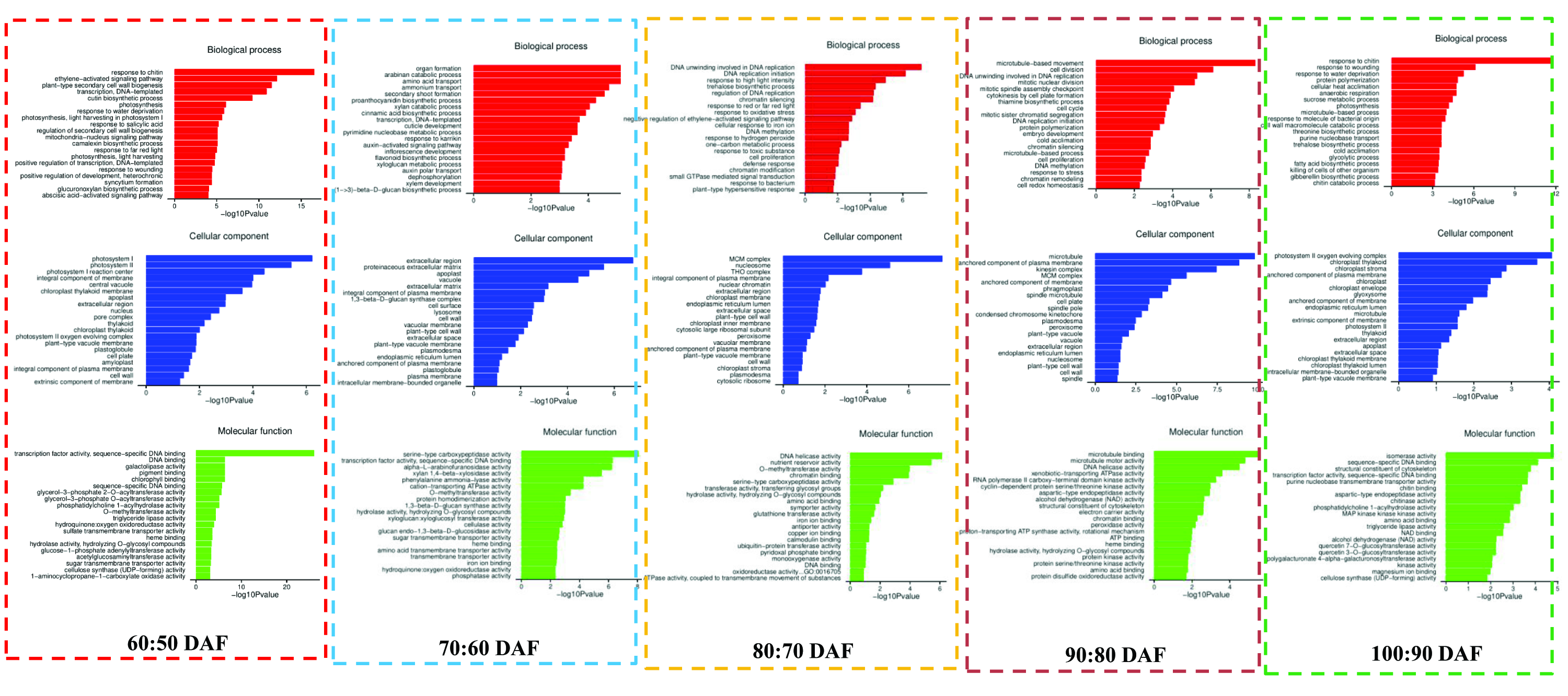

Supplement: Supplementary file 7 — Supplementary Figure S3. [file 41598_2021_82130_MOESM7_ESM.tif]

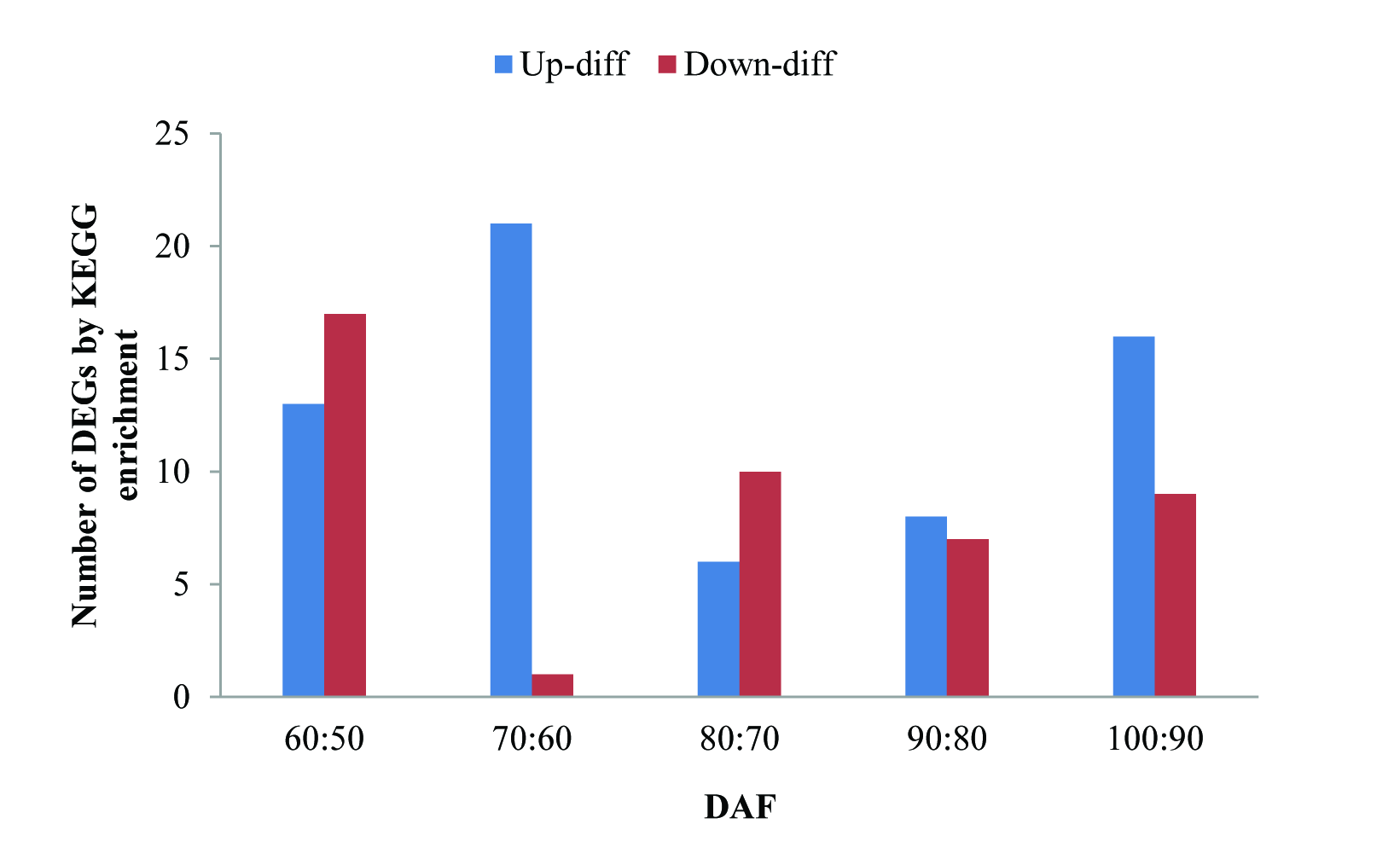

Supplement: Supplementary file 8 — Supplementary Figure S4. [file 41598_2021_82130_MOESM8_ESM.tif]
